# Health Departments in a Brave New World

**DOI:** 10.5888/pcd10.130003

**Published:** 2013-03-21

**Authors:** Christopher Maylahn, David Fleming, Guthrie Birkhead

**Affiliations:** Author Affiliations: David Fleming, Public Health Seattle and King County, Seattle, Washington; Guthrie Birkhead, New York State Department of Health and University at Albany School of Public Health, Albany, New York.

The number of people at risk for chronic diseases is increasing, and methods for reducing risk and promoting health are becoming more complex. Demands of changing political and social environments, as well as economic and demographic trends, are forcing state and local health departments to reassess what is most important and make judicious choices that will yield the greatest gains. Health departments cannot afford to squander time and resources on ineffective programs and policies; to maintain their relevance, they must adopt a public health agenda that is both ambitious and grounded in science.

The mission of health departments is the same today as it was in the 1800s: to ensure conditions in which people can be healthy. As essential players in the nation’s public health system, health departments are operating in the best of times and the worst of times. On the one hand, prevention is getting more attention, and new cross-sector collaborations to embed health in all policies can affect upstream health determinants and push the clinical care system toward more effective, efficient, and equitable care. On the other hand, resources are tight, additional budget cuts loom, and public health is caught up in the larger issues of increasing political polarization and distrust of government. Aligning health with community and economic development can help mitigate these problems. Working with partners from different sectors is essential in reshaping commercial and government policies and practices that drive poor population health (1).

State and local health departments must capitalize on opportunities that now exist to transform the way they do business. Improving the health of Americans requires improvement of population health at multiple levels. The momentum of health care reform and the increased support for prevention must be harnessed. A coordinated approach to prevent chronic diseases and promote health is now supported by new funding provided by the Centers for Disease Control and Prevention (CDC). State public health chronic disease prevention and control programs, especially those that focus on common risk factors such as poor nutrition, low levels of physical activity, and tobacco use, have demonstrated the potential benefits of an integrated model and are a key link to improving our nation’s health (2). State chronic disease directors must expand the effects of local and community-based health programs through coordination and dissemination of evidence-based interventions. Strong leadership from state health directors can reinvent the way health departments operate and stimulate the public health system to be more effective.

We offer 3 priority areas where action can lead to real change in health department programs and practices. Concerted efforts in these areas could substantially improve the health of Americans.

## Defining a “Minimum Package” of Chronic Disease Public Health Services

Chronic diseases cause most illness and deaths in the United States (3), and their treatment is a primary driver of rising health care costs. Preventing or alleviating chronic diseases is the work that public health practitioners face. Yet today, chronic disease prevention programs are neither standardized across public health jurisdictions, nor are they comprehensive. Furthermore, many state and local chronic disease programs have been reduced or eliminated — perhaps on the basis of the “last in, first out” principle — during the years of health department budget cuts caused by the latest economic recession (4). Health care consumers would never consider a health insurance policy that covered heart disease but not cancer or diabetes but not asthma; however, as a nation, we have been accepting of exactly this limited type of coverage from public health.

It is time to shed this complacency. A recent Institute of Medicine (IOM) report (5) called for the definition of a “minimum package of public health services” that should be available to every US resident. This package is not about dictating, in cookie-cutter style, the only chronic disease programs that should be available in a jurisdiction; it is about defining the core chronic disease competencies and capabilities that should be universally available for our public health system to work anywhere. In many ways, chronic disease prevention programs are the poster child for why this proposed change in the way we think about public health services makes sense. Although tobacco use and obesity are the leading actual causes of death in the United States, community prevention programs in one or both areas are unavailable to many of our country’s residents. Furthermore, in most jurisdictions, key capabilities like assessment, policy decision support, and community mobilization are incompletely funded on an ad hoc basis by time-limited categorical resources.

As with all IOM recommendations, defining this “minimum package” will happen only if someone takes it on. Chronic disease public health researchers and practitioners seem to be best able to develop a practical, effective, evidence-based package that allows for enhanced customization according to local surveillance and community needs. The concept of a minimum package has the greatest value if its definition is national in scope. As such, the convening of this process seems like a natural leadership role for CDC, with other federal agencies also contributing.

In short, chronic diseases arise from underlying risk factors only over many years. Health insurers recognize that clinical preventive services yield a much better and more predictable return if everyone provides them. Given the growing mobility of our population, this same logic increasingly applies to community-based chronic disease prevention efforts. We as public health practitioners need to clearly define our roles in designing public health chronic disease prevention programs to work as they should in every community.

## Enhancing Surveillance for Chronic Diseases

A second priority area where action can improve the practice of health department chronic disease programs is public health surveillance, one of the traditional roles of public health. It is implicit in the core function of assessment as articulated by the IOM (6) and is the first of the 10 Essential Public Health Services: “to monitor health status to identify and solve community health problems” (7). Public health surveillance began with communicable diseases, but chronic disease surveillance expanded dramatically in the 1990s and 2000s. It now focuses on monitoring chronic diseases and their precursors in the population by examining data from large information systems such as vital records for cause of death; hospital discharge data for causes of hospitalization*,* particularly preventable hospitalizations; and population-based telephone surveys such as the Behavioral Risk Factor Surveillance System, conducted by all states and some large cities (8). These data sources have been fruitful in tracking chronic diseases such as coronary artery disease, stroke, and diabetes and the effects of tobacco use in the adult population. However, each source has limitations in the timeliness and detail of information and in the ability to measure the precursors of chronic diseases where preventive interventions could be directed. The impending revolution in electronic health information and its potential to better monitor population health provides an opportunity to greatly improve chronic disease surveillance for health department programs. Chronic disease practitioners need to use existing and potential electronic data sources innovatively to best leverage this electronic revolution to improve population health.

Chronic disease programs can improve surveillance by making better use of existing electronic health data and exploring new data sources. For example, information on weight status of the preschool population for obesity surveillance can be provided by electronic data from the Special Supplemental Nutrition Program for Women, Infants, and Children and can fill a gap in public health surveillance for this important population (9). To address a lack of data in the school-aged population, New York mandated that body mass index and weight status be added to required school health physical examinations in certain grade levels. Data are reported electronically from schools over the same Web-based system that collects required school immunization data aggregated by grade level, sex, and school district (10).

The next frontier in chronic disease surveillance is health departments’ use of electronic health record (EHR) data from clinical care systems. For example, an idea that arose from the national “Million Hearts” campaign (11) is to focus on control of hypertension, a major risk factor for heart disease and stroke. Blood pressure is a Stage 1 Meaningful Use objective in the Centers for Medicare and Medicaid Services EHR Incentive Programs (12). If blood pressure measurements and their recording in EHRs can be standardized, the proportion of people with high blood pressure who are undiagnosed or diagnosed but not adequately treated could be determined for the population at the provider, local, state, and federal levels. This determination could support quality-of-care improvement programs in clinical care settings that are linked to community actions that result in better access to physical activity opportunities and healthful foods. This use of EHRs for both public health surveillance and improved clinical care presents the perfect opportunity for public health and clinical medicine to work together to achieve a common goal. As providers of clinical care, health departments should work to ensure that clinical and public health goals are met. Public health practitioners should also help develop meaningful-use objectives and standards.

In many jurisdictions, health departments may be the only entities with the legal authority to aggregate surveillance data at the population level and develop the partnerships necessary to conduct such a coordinated effort. Health departments can also use other types of surveillance of community health determinants such as the availability of healthful foods and opportunities for physical activity. Combining these new sources of electronic health information with innovative approaches to surveillance of the living environment in communities could open new areas of public health action.

There are challenges in realizing this vision of a new public health surveillance system for chronic disease prevention and control. Data standards must be agreed on not only in public health practice but also in curative medicine. Health departments must do a better job of providing surveillance data, distilled to “information for action,” to educate and guide both the clinical and broader communities to prevent and control chronic diseases. The recent requirements of the Affordable Care Act that hospitals develop a community health needs assessment with input from public health practitioners is an ideal opening for state and local health departments to demonstrate the value of surveillance data (13). Finally, the public health workforce will need to adapt to the electronic health revolution by acquiring new, interdisciplinary skills in the areas of informatics, information technology, and quality improvement.

## Increasing the Use of Evidence-Based Decision Making

We certainly do not have all the answers about what works to improve health and prevent disease. However, evidence-based interventions are still underused in many areas of public health practice (14,15). Through research summaries published in the *Guide to Community Preventive Services* (16) and other reviews, evidence is available to act decisively and select proven interventions rather than untested strategies that may waste resources or, worse, do harm. Kohatsu et al defined this as a process of integrating science-based interventions with community preferences to improve the health of populations (17).

Knowledge is emerging in many areas, especially from the steady growth of practice-based research, about how to implement proven interventions and reduce health disparities. This cutting-edge research builds on the suggestion that if we want more evidence-based practice, we need more practice-based evidence (18). States will need a robust evaluation, research, and development capacity to improve their effectiveness at addressing threats to health, learning quickly whether their innovations are working, and demonstrating accountability for their investments (Glen Mays, PhD, MPH, written communication, November 2011). Practice-based research networks supported by the Robert Wood Johnson Foundation (19) and CDC-funded prevention research centers (20) can provide capacity in the design and conduct of this research. Health departments, among others, should be integrally involved with these research partners so that research is embedded in practice innovations, producing evidence for translation of effective strategies. This will help states learn from their successes and failures and refine their efforts. Having more strategies would make a real difference for state and local public health practitioners. The new grants to state health departments for performance management will also help them build capacity more quickly.

Evidence-based decision making requires leadership, an informed workforce, data, and a culture that values the application of science to the planning and implementation of public health programs. A willingness to partner with public health services and systems researchers to learn how evidence can strengthen practice is also essential.

## Conclusion

Defining the minimum package of chronic disease services, improving surveillance, and increasing the use of evidence-based practices are actions we must take. Effecting change will not be easy; it requires leadership, willingness to leave behind familiar ways of doing business, building and strengthening new partnerships, abandoning some efforts, and trying things where we have less experience. The challenge is even greater given the fiscal state of most health departments. However, opportunities do exist and should be pursued. We hope that a larger portion of the funding for health care reform can be allocated to public health agencies. For that to happen, we need to make good use of the resources and do a better job demonstrating the value of public health in measuring and improving the population’s health.
